# Comparative Effectiveness of eConsent: Systematic Review

**DOI:** 10.2196/43883

**Published:** 2023-09-01

**Authors:** Edwin Cohen, Bill Byrom, Anja Becher, Magnus Jörntén-Karlsson, Andrew K Mackenzie

**Affiliations:** 1 AstraZeneca BV The Hague Netherlands; 2 Signant Health London United Kingdom; 3 Oxford PharmaGenesis Oxford United Kingdom; 4 AstraZeneca Gothenburg Sweden; 5 Nottingham Trent University Nottingham United Kingdom

**Keywords:** acceptability, clinical trial, comprehension, digital consent, eConsent, effectiveness, electronic consent, informed consent form, patient engagement, usability

## Abstract

**Background:**

Providing informed consent means agreeing to participate in a clinical trial and having understood what is involved. Flawed informed consent processes, including missing dates and signatures, are common regulatory audit findings. Electronic consent (eConsent) uses digital technologies to enable the consenting process. It aims to improve participant comprehension and engagement with study information and to address data quality concerns.

**Objective:**

This systematic literature review aimed to assess the effectiveness of eConsent in terms of patient comprehension, acceptability, usability, and study enrollment and retention rates, as well as the effects of eConsent on the time patients took to perform the consenting process (“cycle time”) and on-site workload in comparison with traditional paper-based consenting.

**Methods:**

The systematic review was conducted and reported in accordance with the PRISMA (Preferred Reporting Items for Systematic Reviews and Meta-Analyses) guidelines. Ovid Embase and Ovid MEDLINE were systematically searched for publications reporting original, comparative data on the effectiveness of eConsent in terms of patient comprehension, acceptability, usability, enrollment and retention rates, cycle time, and site workload. The methodological validity of the studies that compared outcomes for comprehension, acceptability, and usability across paper consent and eConsent was assessed. Study methodologies were categorized as having “high” validity if comprehensive assessments were performed using established instruments.

**Results:**

Overall, 37 publications describing 35 studies (13,281 participants) were included. All studies comparing eConsenting and paper-based consenting for comprehension (20/35, 57% of the studies; 10 with “high” validity), acceptability (8/35, 23% of the studies; 1 with “high” validity), and usability (5/35, 14% of the studies; 1 with “high” validity) reported significantly better results with eConsent, better results but without significance testing, or no significant differences in overall results. None of the studies reported better results with paper than with eConsent. Among the “high” validity studies, 6 studies on comprehension reported significantly better understanding of at least some concepts, the study on acceptability reported statistically significant higher satisfaction scores, and the study on usability reported statistically significant higher usability scores with eConsent than with paper (*P*<.05 for all). Cycle times were increased with eConsent, potentially reflecting greater patient engagement with the content. Data on enrollment and retention were limited. Comparative data from site staff and other study researchers indicated the potential for reduced workload and lower administrative burden with eConsent.

**Conclusions:**

This systematic review showed that compared with patients using paper-based consenting, patients using eConsent had a better understanding of the clinical trial information, showed greater engagement with content, and rated the consenting process as more acceptable and usable. eConsent solutions thus have the potential to enhance understanding, acceptability, and usability of the consenting process while inherently being able to address data quality concerns, including those related to flawed consenting processes.

## Introduction

### Background

Informed consent to participate remains a fundamental aspect of ethical clinical research. Potential participants of a clinical trial must be given adequate information about the study before they decide whether to participate in accordance with good clinical practice quality standards [1]. Providing informed consent means to agree to take part in the trial and to have understood what is involved, including the risks and benefits of participation [1]. Traditionally, the trial information is conveyed using printed documents that potential participants read before signing to indicate their consent to participate. The informed consent form (ICF), and the associated effective communication of study information, remains among the most challenging and complex processes within the clinical trial landscape. ICFs are known to have poor readability and take too long to be understood and digested effectively [2]. A review of ICFs developed for use in phase III oncology clinical trials showed that these were, on average, 21.4 pages long and that many participants had only a poor understanding of the key elements of their trial [2]. Poor understanding of the study requirements and treatment has been cited as a reason for early withdrawal from clinical trials [3]. To ensure that potential trial participants fully comprehend the study information, ICFs need to convey complicated and technical information in a way that meets the target group’s health literacy capabilities. ICFs have to maintain readers’ engagement sufficiently to ensure that they can make a fully informed decision on whether to participate.

In addition to patient-centered challenges of the ICF process, administrative aspects of the consenting process can pose challenges to investigators conducting clinical trials. Flawed informed consent processes are listed within the top 10 cited regulatory deficiencies and audit findings and are the third highest reason for US Food and Drug Administration (FDA) warning letters to clinical investigators [4-6]. Informed consent was among the top 2 most frequently observed issues in a recent auditing case study, conducted across 37 centers, and problems were identified related to processing errors and missing operational records [7]. Findings included missing signatures, incomplete ICFs, signing of incorrect ICF versions, and unauthorized site staff obtaining consents [7]. These are serious issues that can undermine the integrity of the consent process and the study, and they can result in the inability of researchers to analyze and report the data as intended.

Electronic consent (eConsent) uses digital technologies to enable the consenting process. Components can include multimedia to complement text-based content; interactivity (eg, to handle questions, test knowledge, explain definitions, and allow patients to resume the process from where they left off); electronic signature capture; status dashboards; and version control technology. eConsent aims to improve participant comprehension and engagement with study information and to address data quality concerns that may limit study integrity [8,9]. Although eConsent has been in use for about 15 years, its adoption has been slow until recently, when its accelerated uptake has been driven primarily by the COVID-19 pandemic [9]. Much of the supporting information on the promised benefits of eConsent comes from informal commentaries and reports from eConsent solution providers. In addition to digital technology, and just as with paper-based ICFs, eConsent solutions require good content to be effective. Similar to the computing analogy of “garbage in, garbage out,” poor eConsent content will result in poor overall effectiveness in terms of patient comprehension, acceptability, and usability, irrespective of the quality of the delivery technology.

### Objective

The aim of our systematic review of peer-reviewed research was to provide a summary of qualitative and quantitative evidence to draw conclusions on the relative effectiveness of eConsent in comparison with traditional paper-based consenting.

## Methods

### Literature Searches

The systematic literature review was conducted and reported in accordance with the PRISMA (Preferred Reporting Items for Systematic Reviews and Meta-Analyses) guidelines [10]. A completed PRISMA checklist is included in Multimedia Appendix 1 [10]. We systematically searched the peer-reviewed literature for full papers and conference abstracts relevant to our review using Ovid Embase and Ovid MEDLINE on November 11, 2021. Ovid MEDLINE is equivalent in content to PubMed and additionally includes advanced search options (eg, adjacency operator and within-phrase wildcard) [11,12]. The search string contained terms related to electronic and consenting as follows: ([dynamic OR electronic OR interactive OR multimedia OR online OR tablet OR computer OR digital OR virtual] ADJ4 [consent* OR econsent OR e-consent]). Terms related to “electronic” were limited to the title, abstract, and keywords of a publication. The operator “ADJ4” was used to identify “electronic”- and “consent”-related terms separated by ≤3 words to filter for literature relevant to this review. The records were screened and selected based on our review of the title, abstract, and full text. No language restrictions or publication date limits were applied. The review was not registered, and a protocol was not prepared.

### Inclusion and Exclusion Criteria

Publications reporting original, comparative data on the effectiveness of eConsent in terms of patient comprehension, acceptability, and usability were eligible for inclusion. Comparative data on the effect of eConsent on clinical study enrollment and retention rates, cycle time (ie, time taken to consent), site workload, and stakeholder views were also considered relevant. Head-to-head comparisons of paper-based methods versus eConsent were of particular relevance. Publications that did not present original data (eg, reviews, editorials, and commentaries) were excluded.

### Study Selection

Following the systematic literature searches, duplicate records were removed using the deduplicate option in Ovid. All the remaining records were exported to EndNote X9 (Clarivate), and further duplicates were identified and removed manually. Two reviewers (EC and BB) independently assessed the systematic literature search results and the corresponding full texts following the initial screening of titles and abstracts, with one reviewer (AB) excluding the ineligible publications (eg, reviews), and the team of 3 reviewers resolved any disagreements by consensus-based discussions. Reasons for exclusion and inclusion were captured.

### Data Collection and Summary

Data extraction (conducted by AB and reviewed by BB) included measures and outcomes for patient comprehension, acceptability, usability, enrollment rates, retention rates, cycle time, site workload, and stakeholder views. The extracted data were summarized descriptively. Data on patient comprehension, acceptability, and usability with eConsent versus paper-based ICFs were tabulated as part of the main descriptive summary. An overview of all the studies identified for inclusion is provided in the Multimedia Appendix 2 [13-49].

### Study Categorization

For studies comparing patient comprehension, acceptability, and usability with eConsent versus paper-based ICFs, we estimated the quality of the evidence by categorizing their methodological validity as “high,” “moderate,” or “limited.” The study methodologies that we categorized as having high validity (score=+++) were those that used comprehensive assessments including detailed and open-ended questions (eg, “Tell me what will be done during the study visits”), possibly using established instruments as part of the formal assessments. Methodologies that involved self-rating by participants (eg, “Did you understand the following aspects of the study?”), without formal testing, were categorized as having moderate validity (score=++). When a methodology that involved limited questioning was used in the studies or when methodological details were not reported, we categorized these studies as having limited validity (score=+).

## Results

### Overview

**Figure 1:**
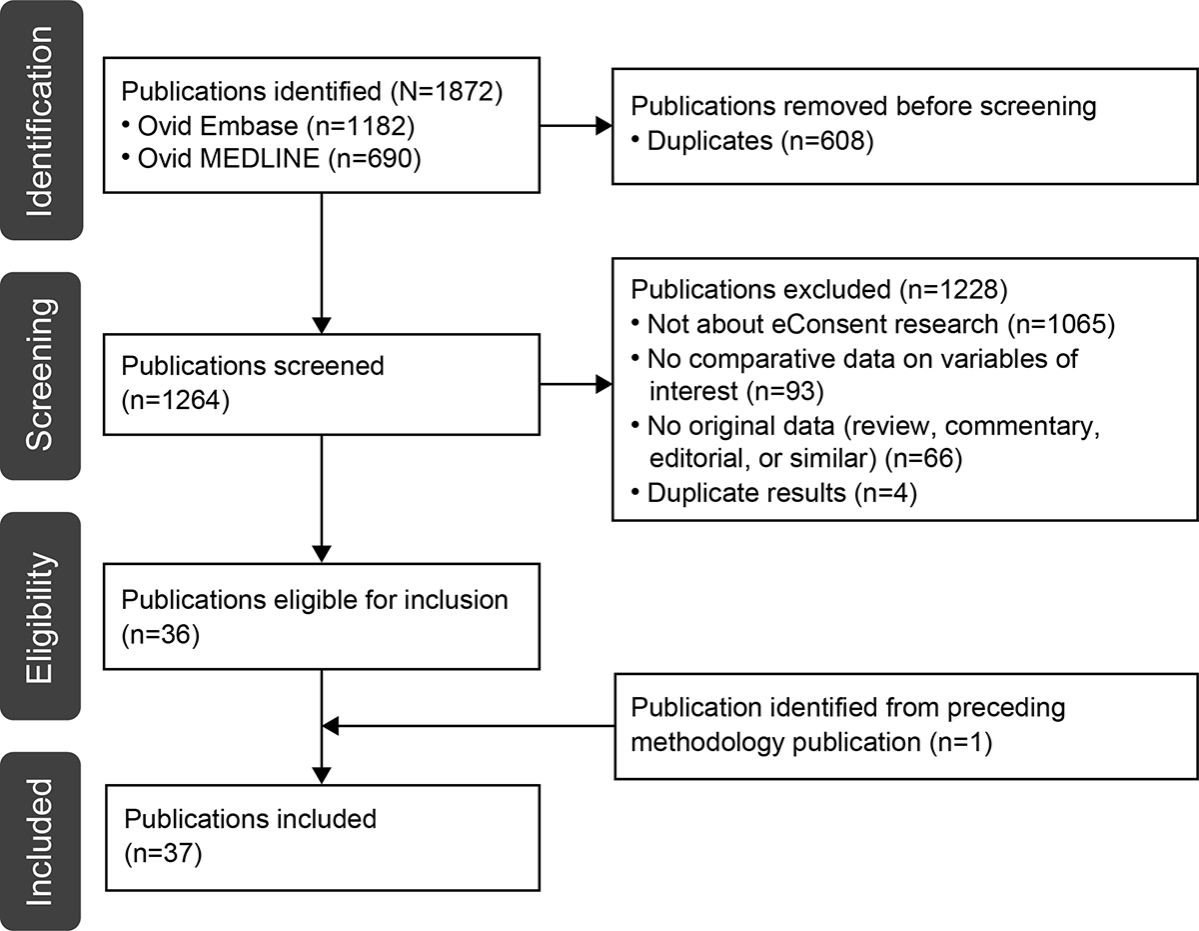
PRISMA (Preferred Reporting Items for Systematic Reviews and Meta-Analyses) flowchart of the systematic literature search. eConsent: electronic consent.

The systematic literature search identified 1872 publications (Figure 1). Of these 1872 publications, 608 (32.48%) duplicates were excluded before screening, and a further 1228 (65.6%) were excluded based on screening by title, abstract, and full publication, with the most common reason for exclusion being that the publication did not report on eConsent research. A total of 36 studies met the eligibility criteria [13-48], and an additional outcomes publication [49] was retrieved manually based on the identification of its accompanying methodology article during screening. Thus, in total, 37 publications (32 full publications and 5 conference abstracts) were included in this review (Multimedia Appendix 2) [13-49].

The included publications together described 35 studies (2 studies were each covered by 2 publications). Most of the studies (28/35, 80%) were from North America (United States: n=26, 93%; Canada, n=2, 7%; Multimedia Appendix 2). The remaining studies (7/35, 20%) were from Europe (Italy: n=1, 3%; Ireland and United Kingdom: n=1, 3%; United Kingdom: n=1, 3%), Australia (n=2, 6%), and Gambia (n=1, 3%), and 1 (3%) was multinational. Taken together, these studies included a total of 13,281 participants. The number of participants per study ranged from 9 to 3485. In total, 13 (37%) out of 35 studies were conducted as part of randomized (n=10) or nonrandomized (n=3) clinical research studies, 14 (40%) studies were simulated consent studies, and 8 (23%) studies were survey or interview studies. Most of the research and simulation consent studies (23/27, 85%) were conducted in person (Multimedia Appendix 2). Comparative data on patient comprehension, acceptability, and usability with eConsenting were provided in 26, 13, and 6 studies, respectively, of which 20, 8, and 5 studies included comparisons for eConsent versus paper-based ICFs, respectively. Aspects of eConsent in relation to enrollment rates, retention, cycle time, staff workload, and stakeholder views were covered in 12, 1, 13, 3, and 5 studies, respectively. Age groups ranged from 8 years to 91 years in the 14 studies that included age range information. Among the 23 studies that provided sufficient information on average (mean or median) age, the average age was <50 years in 12 studies and ≥50 years in 11 studies.

### Patient Comprehension

Overall, 26 studies (8778 participants in total) assessed the aspects of patient comprehension with eConsenting.

#### Patient Comprehension: eConsenting Versus Paper

Comparative information on comprehension with eConsenting versus paper-based ICFs was provided in 20 studies, including a total of 6769 participants (of whom 5809 participants contributed comparative data on comprehension; Table 1). All 20 studies reported significantly better understanding with eConsent, better understanding but without significance testing, or no significant differences in overall understanding (Table 1).

**Table 1:** Studies providing comparative findings on comprehension with electronic consent (eConsent) versus paper informed consent form.

Study, year	Participants		Methodology		Comprehension findings^a^
	Sample size, N	Age (years)	Measure	Validity^b^	
Abujarad et al [13], 2021	50	eConsent: mean 47 (SD 15; range NR^c^) Comparator: mean 38 (SD 15; range NR)	QuIC^d^ Self-rating, not tested	QuIC: +++ Self-rating, not tested: ++	QuIC: no significant difference Self-rating, not tested: significantly better scores with eConsent vs paper for 2 of 4 informed consent–related concepts (*P*=.02; *P*=.045); other 2 concepts not significantly different
Afolabi et al [14], 2015	311	Mean NR, (SD NR; range NR); >90% aged 18-49	DICCQ^e^	+++	Better scores with eConsent vs paper and verbal. Differences significant on 3 of 4 d tested: day 0 (*P*=.04), day 14 (*P*=.04), and day 21 (*P*=.04)
Bickmore et al [15], 2009	29	Mean 60 (SD NR; range 28-91)	BICEP^f^	+++	Better scores with eConsent vs paper or verbal. Significantly different scores between paper, verbal, and eConsent (*P*=.006)
Buckley et al [16], 2020	97	NR	10 questions (details NR)	+	Better scores with eConsent vs paper for both study protocols tested. Difference significant for 1 protocol (“genomic”; *P*<.01)
Chalil Madathil et al [18], 2013	40	Mean NR (SD NR; range 18-77)	7 questions (complicated language)	+	Study researchers report that results suggested better understanding with iPad vs paper (full details NR)
Chapman et al [19,20], 2021 and 2020	298	Mean 63 (SD 8; range 45-74)	5 true or false questions	+	Significantly better scores with eConsent vs paper for question on participation requirements (*P*<.001) and data sharing (*P*=.03); no significant differences for other 3 questions
Harmell et al [25], 2012	35	Outpatients eConsent: mean 57 (SD 10) Comparator: mean 57 (SD 10) Healthy individuals eConsent: mean 49 (SD 16) Comparator: mean 53 (SD 12; ranges NR)	UBACC^g^ MacCAT-CR^h^	UBACC: +++ MacCAT-CR: +++	UBACC Outpatients: significantly better scores with eConsent vs paper (*P*=.03; Cohen d=0.94) Healthy individuals: no significant difference MacCAT-CR Outpatients: no significant differences Healthy individuals: no significant differences
Jayasinghe et al [27], 2019	35	Focus group: 77 (SD 8; range NR) Pilot: 75 (SD 7; range NR)	UBACC	+++	Better scores with eConsent vs paper at baseline and week 1, but effect not statistically significant (*P*=.50; Hedges g=0.30)
Jeste et al [28], 2009	60	Paper: mean 54 (SD 9) Multimedia: mean 55 (SD 7)	UBACC MacCAT-CR	UBACC: +++ MacCAT-CR: +++	UBACC Outpatients: significantly better scores with eConsent vs paper (*P*<.001; 95% CI 0.59-0.77) Healthy individuals: no significant difference MacCAT-CR Outpatients: better scores with eConsent vs paper for all 4 concepts. Differences significant for understanding (*P*=.006; 95% CI 0.54-0.74) and choice (*P*=.02; 95% CI 0.51-0.57) Healthy individuals: significantly better scores with eConsent vs paper for understanding (*P*=.02; 95% CI 0.52-0.79); no significant difference for other 3 concepts
Knapp et al [49], 2021	109	Median 13 (range 11-14)	Custom survey (DMQ^i^; 9 questions)	++	Significantly better scores with eConsent vs paper for understanding (*P*=.003) and confidence in decision-making (*P*=.04); no significant differences for other 7 questions or in total scores
McCarty et al [30], 2015	56	Mean 73 (SD NR; range 55-86)	Custom survey after 6 mo (38 questions)	++	No significant differences for 36 of 38 questions. Significantly better scores with eConsent vs paper for 2 knowledge questions (both *P*<.05)
McGraw et al [32], 2012	43	Mean 38 (SD NR; range 18-68)	Interviews	+	No difference in the proportion of participants recalling concepts spontaneously
Rothwell et al [35], 2020	669	Mean 30 (SD 5)	QuIC parts A and B	+++	Video versus paper: significantly better scores with video for knowledge (*P*<.001) and understanding (*P*=.003) Interactive app versus paper: significantly better scores with app for knowledge (*P*=.003); no significant difference for understanding
Rothwell et al [36], 2014	62	NR	Custom survey (14 questions)	++	Significantly better scores with eConsent vs paper for 4 of 14 questions (*P*=.047; *P*=.002; *P*<.001; *P*=.02); no significant differences for other 10 questions
Rowbotham et al [37], 2013	75	Mean 50 (SD NR; range 18-80)	Custom survey (12 questions)	++	Significantly better scores with eConsent vs paper (*P*<.001)
Simon et al [39], 2016	200	Mean 47 (SD NR; range 18-86)	QuIC parts A and B	+++	eConsent improved understanding vs paper (*P*=.04; partial η2=0.021); no difference in confidence of understanding
Simon et al [41], 2021	501	Mean 47 (SD N; range 18-84)	QuIC parts A and B	+++	No difference overall in understanding. Confidence in understanding was significantly lower with eConsent than paper (*P*=.02)
Sonne et al [42], 2013	61	Mean 43 (SD 14; range NR)	Custom survey (20 questions)	++	No significant differences
Varnhagen et al [45], 2005	3045	Mean NR (SD NR; range NR); 84% aged ≥45	Unprompted recall	+	No significant differences
Warriner et al [47], 2016	33	eConsent: mean 69 (SD 7) Paper: mean 71 (SD 9; range NR)	Health-ITUES^j^, QuIC	+++	No significant differences

^a^Significant *P* values, effect sizes, and CIs are reported when provided in the publications.

^b^Methodological validity was categorized as “high” (+++), “moderate” (++), or “limited” (+).

^c^NR: not reported.

^d^QuIC [50]: Quality of Informed Consent. Part A=20 questions self-rated (agree, unsure, and disagree); part B=14 questions to self-rate the understanding of different aspects on a scale of 1 to 5.

^e^DICCQ [51]: Digitized Informed Consent Comprehension Questionnaire. A total of 26 questions (9 yes or no, 6 multiple-choice single answers, 4 multiple-choice multiple answers, and 7 verbal recall) and investigator-rated responses.

^f^BICEP [52]: Brief Informed Consent Evaluation Protocol. Contains 12 open questions, scored by the interviewer and assesses pressure to participate, understanding of care if not consented, benefits, risks, study requirements, purpose of study, when the study ends, and when participants could withdraw consent.

^g^UBACC [53]: University of California San Diego Brief Assessment of Capacity to Consent. It contains 10 open questions on study purpose, requirement to participate, impact of withdrawing, study requirements, risks and benefits, and costs.

^h^MacCAT-CR [54]: MacArthur Competence Assessment Tool for Clinical Research. Understanding (scores range from 0 to 26), Appreciation (0-6), Reasoning (0-8), and expression of a choice.

^i^DMQ: Decision-Making Questionnaire.

^j^Health-ITUES: Health Information Technology Usability Evaluation Scale.

Different methods were used across studies to assess comprehension, some of which were more robust than others in their approaches. Overall, 10 studies included established instruments to assess comprehension, and their methodological validity was thus categorized as “high” (score=+++) [13-15,25,27,28,35,39,41,47]. The instruments used included the Brief Informed Consent Evaluation Protocol [15,52], Digitized Informed Consent Comprehension Questionnaire [14,51], MacArthur Competence Assessment Tool for Clinical Research [25,28,54], Quality of Informed Consent [13,35,39,41,47,50], and University of California San Diego Brief Assessment of Capacity to Consent [25,27,28,53].

Overall, 60% (6/10) of the “high” validity studies reported significantly better understanding with eConsent than paper-based ICFs for at least some of the concepts assessed using established instruments, with no statistical tests in favor of the paper process [14,15,25,28,35,39]. The remaining 4 (40%) of the 10 studies reported no significant difference in comprehension between eConsent and a paper-based consent process [13,27,41,47], with 1 study reporting statistically nonsignificant better comprehension using eConsent [27]. However, confidence in understanding was significantly lower with eConsent than with paper-based ICFs in 1 study that observed no difference in overall understanding [41].

Furthermore, 6 studies included custom surveys or participant self-rating without formal testing to evaluate comprehension, and their methodological validity was thus categorized as “moderate” (score=++) [13,30,36,37,42,49]; one of these studies used both “high” and “moderate” validity methodologies [13]. Of the 6 “moderate” validity studies, 67% (n=4) of studies reported significantly better comprehension with eConsent than with paper-based ICFs for at least some of the concepts assessed [13,36,37,49], with the remainder reporting no significant differences [30,42].

The remaining 5 studies (covered by 6 publications) used limited questioning or did not report methodological details, and their methodological validity was thus categorized as “limited” (score=+) [16,18-20,32,45]. Of the “limited” validity studies, 3 (covered by 4 publications) reported better comprehension with eConsent than with paper-based ICFs for at least some aspects [16,18-20], and 2 reported no differences [32,45].

#### Patient Comprehension: Other Evidence

In the study by Rothwell et al [36] (Table 1), participants in the eConsent group were interviewed after the consent process; several noted that the eConsent format was easy to understand and held their attention more than a paper-based approach would have done. Several further studies assessed comprehension either by comparing different electronic formats at baseline versus postconsent time point or by describing results from interviews about patient preferences [22-24,33,34,43,48] (Multimedia Appendix 2). Comprehension was significantly better with a highly interactive eConsent version than with less-interactive versions in a study by Geier et al [22]. Participants in a study by Naeim et al [33] found that information was easier to understand when the video presentation was animated rather than text based. Perrault and Keating [34] found that text layouts using line spacing, bold font, and bullet points could improve comprehension compared with a bullet-pointed flowchart. Golembiewski et al [23] and Harle et al [24] observed no significant differences in understanding between a standard tablet-based version and versions that had key terms hyperlinked to additional research-related information. Tait et al [43] showed that parents’ and children’s understanding of clinical trial–related terminology was improved after eConsenting compared with baseline. Most participants (67%) in a survey of clinical trial researchers by Zeps et al [48] thought that eConsent would improve patients’ comprehension.

### Patient Acceptability

Overall, 13 studies (1694 participants in total) assessed the aspects of patient acceptability with eConsenting.

#### Patient Acceptability: eConsenting Versus Paper

Comparative information on the acceptability of eConsenting versus paper-based ICFs was provided in 8 studies, including a total of 631 participants (of whom 621 participants contributed comparative data on acceptability; Table 2). All 8 studies reported significantly higher satisfaction or enjoyment with eConsent, higher satisfaction but without significance testing, or no differences in acceptability (Table 2). Only one of the studies was categorized as having “high” methodological validity, having used an established instrument to assess acceptability, in this case, the Computer System Usability Questionnaire [18,55]. This study reported statistically significant higher satisfaction scores with eConsent compared with paper-based ICFs [18]. The methodology used to assess acceptability was categorized as having “limited” validity in the remaining 7 studies (covered by 8 publications) [13,15,19,20,25,37,42,47]. Furthermore, 6 studies with “limited” validity reported higher acceptability with eConsent than with paper-based ICFs [13,15,25,37,42,47], and in 3 of these studies, at least some of the differences were statistically significant [13,15,37].

**Table 2:** Studies providing comparative findings on acceptability of electronic consent (eConsent) versus paper informed consent form.

Study, year	Participants			Methodology			Acceptability findings
	Sample size, N	Age (years)	Measure		Validity^a^		
Abujarad et al [13], 2021	50	eConsent: mean 47 (SD 15; range NR^b^) Comparator: mean 38 (SD 15; range NR)	3 questions as part of a 12-question survey (Likert scale)		+	Significantly higher satisfaction scores with eConsent vs paper for 1 of 3 questions (*P*=.01); no significant differences for other 2 questions	
Bickmore et al [15], 2009	29	Mean 60 (SD NR; range 28-91)	1 question (Likert scale)		+	Significantly higher satisfaction scores with eConsent vs paper or verbal (*P*=.02)	
Chalil Madathil et al [18], 2013	40	Mean NR (SD NR; range 18-77)	CSUQ^c^ overall satisfaction score		+++	Higher satisfaction scores with eConsent formats vs paper. Difference across the different formats statistically significant (*P*<.05)	
Chapman et al [19], 2021; Chapman et al [20], 2020	298	Mean 63 (SD 8; range 45-74)	3 questions (multiple choice)		+	Similar levels of overall acceptability	
Harmell et al [25], 2012	35	Outpatients eConsent: mean 58 (SD 9) Comparator: mean 57 (SD 10) Healthy individuals eConsent: mean 49 (SD 16) Comparator: mean 53 (SD 12) (ranges NR)	1 question (multiple choice)		+	Proportion of patients preferring current vs past consenting experience higher with eConsent vs paper (*P* value NR)	
Rowbotham et al [37], 2013	75	Mean 50 (SD NR; range 18-80)	2 questions (Likert scales)		+	Significantly higher scores with eConsent vs paper for enjoyment (*P*<.05). No significant difference for satisfaction (*P*=.09)	
Sonne et al [42], 2013	61	Mean 43 (SD 14; range NR)	1 question		+	79% of participants preferred eConsent over paper format	
Warriner et al [47], 2016	33	Tablet: mean 69 (SD 7) Paper: mean 71 (SD 9; ranges NR)	3 questions (Likert scales)		+	Higher satisfaction with eConsent vs paper, but difference not statistically significant	

^a^Methodological validity was categorized as “high” (+++), “moderate” (++), or “limited” (+).

^b^NR: not reported.

^c^CSUQ [55]: Computer System Usability Questionnaire. It contains 19 questions measuring overall satisfaction, system usefulness, information quality, and interface quality.

#### Patient Acceptability: Other Evidence

Several studies described viewpoints regarding consenting format preferences or comparing acceptability when using different electronic formats [23,24,26,29,31,46] (Multimedia Appendix 2). The survey and interview results indicated a preference for eConsent over paper-based ICFs. McGowan et al [31] reported that 52% of their study sample preferred eConsent, 46% had no preference, and only 3% would have preferred face-to-face consenting. Similarly, in a survey by Vercauteren et al [46], 41% of the respondents preferred eConsent, 41% had no preference, and only 16% preferred paper-based ICFs. The focus group participants in a study by Jimison et al [29] thought that eConsent was useful and could replace the paper-based ICFs. Only 22% of legally authorized representatives that eConsented on behalf of clinical study patients would have preferred a paper-based ICF in a study by Haussen et al [26]. The studies by Golembiewski et al [23] and Harle et al [24] compared different formats of eConsenting and observed no significant differences in acceptability between the standard version and the version with hyperlinks to additional materials.

### Patient Usability

Overall, 6 studies (582 participants in total) assessed the aspects of patient usability with eConsenting.

#### Patient Usability: eConsenting Versus Paper

Comparative information on the usability of eConsenting versus paper-based ICFs was provided in 5 studies, including a total of 542 participants (of whom 532 participants contributed comparative data on usability; Table 3). All 5 studies reported significantly better usability with eConsenting, better usability but without significance testing, or no differences in usability (Table 3). One study had “high” methodological validity for assessing usability, having measured this via the Computer System Usability Questionnaire, and reported statistically significant higher usability scores with eConsent than with paper-based ICFs [18]. One study had “moderate” methodological validity and observed no overall significant difference in the usability between eConsent and paper-based ICFs [27]. Three studies (4 publications) with “limited” validity reported better usability with eConsent than with paper-based ICFs [13,19,20,49], and in 2 of these studies, at least some of the differences were statistically significant [13,19,20].

**Table 3:** Studies providing comparative findings on the usability of electronic consent (eConsent) versus paper informed consent form.

Study, year	Participants		Methodology			Findings
	Sample size, N	Age (years)	Measure	Validity^a^		
Abujarad et al [13], 2021	50	eConsent: mean 47 (SD 15; range NR^b^) Comparator: mean 38 (SD 15; range NR)	1 question (Likert scale)	+	eConsent participants scored the process as significantly less difficult than paper consent participants (*P*=.02)	
Chalil Madathil et al [18], 2013	40	Mean NR (SD NR; range 18-77)	CSUQ^c^ system usefulness and interface quality subscales	+++	Higher usefulness and interface quality scores with eConsent formats vs paper. Difference across the different formats statistically significant (*P*<.05)	
Chapman et al [19,20], 2021 and 2020	298	Mean 63 (SD 8; range 45-74)	2 questions (multiple choice) plus successful completion	+	Significantly better scores with eConsent vs paper for engagement with study information (*P*<.001); no significant difference for improvement. All participants successfully completed the consenting process	
Jayasinghe et al [27], 2019	35	75 (SD 7; range NR)	10 questions (Likert scales)	++	Overall, no statistically significant difference with eConsent vs paper	
Knapp et al [49], 2021	109	Median 13, range 11-14	1 question (Likert scale)	+	Better scores with eConsent vs paper (*P* value NR)	

^a^Methodological validity was categorized as “high” (+++), “moderate” (++), or “limited” (+).

^b^NR: not reported.

^c^CSUQ [55]: Computer System Usability Questionnaire. It contains 19 questions measuring overall satisfaction, system usefulness, information quality, and interface quality.

#### Patient Usability: Other Evidence

Participants who were asked about their impressions of the electronic and paper-based informed consent processes described the electronic process as well organized, easy to use, and useful in a study by Simon et al [40] (Multimedia Appendix 2).

### Enrollment Rates

A total of 12 studies (6399 participants in total) assessed the effect of the ICF format on the aspects of patient enrollment, with mixed results. Comparisons of consenting rates with eConsenting versus paper-based ICFs were reported in 5 studies [15,18,28,35,41]. In the study by Bickmore et al [15], a significantly higher proportion of participants in the eConsent group than those in the paper group signed their ICFs (*P*=.01). Consenting rates were also higher with eConsent than with the paper-based ICFs in a study by Chalil Madathil et al [18] (*P* value not reported). Consenting rates were similar between the groups in a study by Jeste et al [28] (*P* value not reported), and Rothwell et al [35] reported that consenting rates were similar in paper-based ICFs and video eConsent groups, but the rates were lower in the app eConsent group (*P* value not reported). In a study by Simon et al [41], enrollment was significantly higher with a face-to-face informed consent process than with eConsenting (*P*=.004), although immediately after the consenting process, similar proportions of the 2 groups had reported their intention to enroll; the eConsent process was conducted at the same location as the face-to-face process.

Overall, 4 studies reported eConsenting rates using different electronic media formats [21,23,24,33,38]. No significant differences in enrollment rates were observed with animated versus text-based video consents by Naeim et al [33], with video- versus text-based consenting by Fanaroff et al [21], or with different levels of eConsent interactivity by Golembiewski et al [23] and Harle et al [24]. Siegel et al [38] observed an increase in enrollment rates after a content redesign and attributed the increased rates to the web-based consenting being directly integrated with new patient on-boarding.

Furthermore, 3 studies described results about preferences and found that the format of the ICF made little difference to participants’ decision-making regarding study participation [13,17,49]. In the study by Abujarad et al [13], participants were asked to score the importance of the consenting process in their decision to participate; scores were not significantly different between the paper ICF and the eConsent groups. In the study by Knapp et al [49], similar proportions of patients in the paper-based ICF and the eConsent groups found that the trial information provided helped them make their decision about whether to take part [49]. Study researchers who were surveyed in the study by Cagnazzo et al [17] thought that the use of eConsent had little influence on whether patients declined to participate in a stud.

### Retention

None of the included studies reported overall study retention comparisons between the 2 consenting approaches of paper-based ICFs versus eConsenting. Fanaroff et al [21] (3485 participants) assessed different formats of eConsenting and found no statistically significant differences in the proportions of enrolled patients who subsequently completed the 2 requested study procedures, namely, a blood draw and survey questions.

### Cycle Time

A total of 13 studies (2063 participants in total) assessed cycle time, and 10 studies (covered in 11 publications) assessed the comparative effect of eConsent versus paper-based ICFs on consenting times, 2 studies (3 publications) asked about perceived consenting time, and 1 study assessed consenting times with different electronic formats. eConsenting took more time than paper consenting in the studies by Chapman et al [19,20] (*P*=.006), Jayasinghe et al [27] (*P*<.001), McCarty et al [30] (*P*<.001), Rowbotham et al [37] (*P*<.001), Simon et al [39] (*P*<.001), Sonne et al [42] (*P* value not reported), and Varnhagen et al [45] (*P*<.001; partial η^2^=0.36). eConsenting was faster than paper consenting in the studies by Afolabi et al [14] and Jeste et al [28] (*P* value not reported in either study). Chalil Madathil et al [18] found no significant effect of consenting condition on time taken to complete the task. Abujarad et al [13] and Warriner et al [47] asked participants about their *perceived* time to complete the task and found no statistically significant differences between the eConsent and paper-based ICF groups. Different electronic formats of eConsenting did not significantly affect consenting times in the study by Golembiewski et al [23] and Harle et al [24].

### Site Workload

In total, 3 studies (3284 participants in total) assessed the site workload. Hospital staff in the study by Chalil Madathil et al [18] reported a less subjective workload with eConsenting than with paper-based formats (*P*=.02), and the responses were assessed using the National Aeronautics and Space Administration Task Load Index. Site advisory group feedback in the study by Vanaken and Masand [44] included a beneficial reduction in the administrative burden and reduction in paper trail, although a potential for increased workload was also noted, for example, in relation to training and device management. In the study by Zeps et al [48], clinical trial researchers noted that eConsent devices could be clunky and prone to malfunction, which increased overall study time and burdened trial staff.

### Stakeholder Views

Overall, 5 studies (3416 participants in total) assessed stakeholder views. Staff in the study by Chalil Madathil et al [18] preferred eConsenting formats over paper-based consenting (differences among systems, *P*<.005). In the study by Warriner et al [47], the findings from a telephone survey of practice sites that administered both consent processes favored eConsent over paper-based ICFs, but the differences were not statistically significant. Health Authority representatives were in favor of the broad implementation of eConsent in alignment with local regulations in the study by Vanaken and Masand [44]. However, approximately half (53%) of the surveyed research participants preferred having both a paper document and an eConsenting system [44]. Similarly, most centers (65%) in a survey by Cagnazzo et al [17] preferred using a paper-based ICF in parallel with eConsenting. Clinical research stakeholders surveyed by Cagnazzo et al [17] in late 2020 thought that at a regulatory level, the use of eConsent might increase the time to study approval. In the survey of clinical trial researchers’ opinions on eConsent conducted by Zeps et al [48] in early 2019, a total of 68% of the respondents believed that ethics committees would not approve the use of eConsent or were unsure if they would, while 67% of the respondents thought that the lack of standardized, consistent guidance across the sector was an important barrier to success and 60% of the respondents believed that the high initial cost might be a barrier to uptake.

## Discussion

### Principal Findings

Our systematic literature review aimed to assess the effectiveness of eConsent in terms of patient comprehension, acceptability, usability, study enrollment and retention rates, cycle time, and site workload, primarily in comparison with traditional paper-based consenting. We identified 37 primary publications for inclusion that together described 35 studies (13,281 participants in total). Our results showed that compared with patients who used paper-based consenting, patients who used eConsent had a better understanding of the trial information, showed greater engagement with content, and rated the consenting process as more acceptable and usable. Cycle times were increased with eConsent, potentially reflecting the greater patient engagement with the content. Data on enrollment, retention, and site workload effects were limited. Some general themes emerged in relation to the effectiveness of eConsent, its administrative aspects, and the variability in eConsenting formats used across studies. We have discussed these under the following subheadings.

### Effectiveness

#### Comprehension, Acceptability, and Usability

Informed consent involves providing potential clinical trial participants with adequate information on what the study involves, including the risks and benefits of participation, to allow them to make a fully informed decision on whether to participate. Knowing that potential trial participants have understood the study information is thus of utmost importance. Our systematic review showed good evidence of improvements in comprehension with eConsent versus paper-based ICFs. Assessments of patients’ experiences with eConsenting need to distinguish between the content of the eConsent information and the workability of the digital platform. Our findings in terms of comprehension, acceptability, and usability were consistent, showing either overall benefits to patients of eConsenting versus paper-based ICFs or no significant overall differences. Patients reported higher satisfaction and enjoyment with the eConsent process than with paper-based consenting and found eConsenting both more useful and less difficult to use than paper versions. None of the studies reported significantly higher overall patient benefits with paper-based ICFs than with eConsent.

Studies were limited in terms of their exploration of why eConsent was more effective than paper-based consenting. Craik and Lockhart [56], in their “levels of processing” framework for memory research, suggest that learning and memory are improved when the information is processed in depth. This deeper level of processing might be achieved in many ways. Research by Dellson et al [57] suggests that use of good graphic design in consent materials, for example, using illustrations rather than text to explain treatment regimens, raises potential participants’ motivation to engage with the materials and facilitates their understanding of the clinical study. In the cognitive theory of multimedia learning, Mayer [58] proposes that people can learn more deeply with multisensory processing, when audio and visual information is presented together at the same time [59,60]. Further improvements in learning efficiency are obtained with user-focused active engagement [61]. Compared with text alone, the use of multimedia is also likely to increase attention arousal [62], which is typically associated with increased learning [63,64]. However, maximizing sustained attention needs to be balanced with the cognitive processing effort, which should not be increased beyond the cognitive capacity of the participant [58]. In their study of web-based lectures, Chen and Wu [65] found that the visual information presented with a voice-over resulted in increased sustained attention workload and negatively affected learning performance, compared with the visual information presented with video and audio of the presenter. Future research might wish to explore the role of cognitive capacity in eConsent comprehension and ways to mitigate cognitive demands, for example, via the use of self-pacing [66].

#### Effectiveness in Older Age Groups

Encouragingly, many studies that we examined included patient groups up to the age of 91 years. Although studies do not examine age cohorts separately, the positive effectiveness findings also applied to patients in older adult age groups, indicating that age does not have a negative impact on the effectiveness of eConsent, although more data for a comprehensive assessment are needed. In a cardiovascular study that included 298 participants with a mean age of 63 (range 45-74) years, those randomized to eConsent, consisting of multimedia including video-, audio-, and computer-based finger-signed consent, had a better understanding of study requirements than their counterparts randomized to the traditional paper-based consenting [19,20]. It has been found that older adults integrate more of the audiovisual information in their environment when performing tasks and benefit more from multisensory processing than younger adults do [67], thus supporting the use of eConsent in older age groups. The perceived lower technology literacy in some older cohorts can be mitigated with a good solution design and effective training [68].

In addition to comprehension, the usability data showed that eConsenters had better engagement with study information than their paper-based ICF counterparts, and acceptability was similar for the 2 consenting formats [19,20]. Focus group discussions with individuals aged ≥65 years yielded frequently cited advantages of eConsenting, including its convenience and the usefulness of additional features such as definitions, graphics, and audio [27]. Although not evaluated here, it is likely that age per se has less of an impact than other patient characteristics, such as cognitive ability, dexterity, and technology literacy, on the usability and acceptability of digital solutions within clinical trials.

#### Enrollment

Overall, there was no consensus across publications as to whether a patient’s likelihood to enroll in a study is affected by whether the consenting process is electronic or on paper [15,18,28,35,41]. When questioned, the patients indicated that the format of the ICF made little difference to their decision-making regarding study participation [13,17,49]. However, eConsenting had the potential to increase patient enrollment by increasing accessibility when integrated into a web-based patient platform [38].

#### Retention

Potentially more relevant than enrollment effects is whether improved patient comprehension of the study and its requirements leads to enhanced trial retention. We identified a marked gap in the comparative research on the effect of eConsent on patient retention within clinical studies. The observed improvements in comprehension with eConsent could potentially be used as a surrogate for retention because we know that not fully understanding the study requirements beforehand is a key reason for early withdrawal from clinical trials [3].

### Administration

#### Cycle Times

Most studies in this review that assessed the time it took for patients to undertake the consenting process with eConsent versus paper found that eConsenting took more time than paper consenting [19,20,27,30,37,39,42,45]. This finding is not unexpected. As eConsent is better able to hold patients’ attention than paper-based approaches [36], eConsenting patients are likely to engage more fully with the information provided, thus increasing cycle time. Explanations provided by the primary study authors for the increased time taken with eConsent included that this format enabled participants to engage more with the study information [19,20], that participants made use of the opportunities to view additional information available in the eConsent format [27], or that participants took time to listen to slide narration [39]. To mitigate the increase in cycle time, clinical researchers might consider providing remote eConsent access ahead of a study visit.

#### Site Workload

We identified only limited comparative information on site workload. None of the studies assessed workload across the entirety of a clinical trial. The workload advantages of the fully digitized consent process versus paper-based consenting may become visible only later in the clinical study timeline, when the administrative burden with paper-based consenting may increase owing to data quality issues.

One study assessed hospital staff’s subjective workload and found it to be reduced with eConsent versus paper [18]. Advisory group feedback included a reduction in administrative burden and paper trail, better version control, fewer issues around missing dates or signatures on forms, improved data quality, better participant oversight, and reduced number of site visits, potentially offset by an increased workload in relation to training and device management [44]. Site staff and health authority representatives tended to prefer eConsenting formats over paper-based consenting [18,44], although a preference for using both a paper document and an eConsenting system was also reported [17,44]. Technical difficulties with devices were noted as a potential burden for trial staff [48]. Sonne et al [42] described 1 in 5 participants with technical difficulties, including videos not loading or needing to be restarted, and internet connection issues, although that study was published in 2013 and is thus unlikely to reflect current setups.

#### Regulatory Aspects

Flawed informed consent processes are among the topmost regulatory and inspection findings for clinical trials [4-7]. Although we did not review, we expect that eConsent would implicitly protect against most of the common reasons for such findings. eConsent solutions prevent lodgment with incomplete information, missing signatures, or signing of incorrect versions and preclude retrospective signing, which cannot be detected or demonstrably proven with paper-based consenting. A fully digital consent process would allow for the evaluation of the success of the consent form content and system and their continuous improvement for patients. With paper-based ICFs, such evaluations would not be possible without having validated questionnaires included in each study. Moreover, the ability to track the withdrawal of a consent on an individual or a sample level benefits patients by ensuring that their data or samples are not used for future research. The industry would benefit by being able to comply with patients’ wishes by not using data or samples outside of the study. Future work will need to evaluate and confirm these benefits.

### Variability

The types of eConsent used varied considerably across the included studies. Formats included straightforward digitization of paper documents, signature management systems, audio- and video-enhanced content, and fully interactive systems. Across formats, active multimedia engagement principles were observed, and significantly improved comprehension was achieved with highly interactive versions compared with less-interactive eConsent versions [22]. Animated video–based information was found to be easier to understand than text-based videos [33]. Even for text-based formats, use of line spacing, bold font, and bullet points could improve comprehension [34]. This variability in format has implications for future work, which might explore the differences and most effective formats further.

There was also variability in terms of how comprehension, acceptability, and usability were assessed. Among the studies that assessed the effectiveness of eConsenting compared with paper-based consenting, half of the 20 studies on comprehension had high methodological validity, but only 1 of the 8 studies on acceptability and 1 of the 5 studies on usability did so.

In its guidance on the use of electronic informed consent, the FDA notes the following:

[Electronic informed consent] may be used to provide information usually contained within the written informed consent document, evaluate the subject’s comprehension of the information presented, and document the consent of the subject or the subject’s legal authorized representative. Electronic processes to obtain informed consent may use an interactive interface, which may facilitate the subject’s ability to retain and comprehend the information [8].

In line with the FDA guidance, we suggest that a consenting format should be referred to as eConsent only if it can support patient engagement using multimedia components (eg, text, graphics, audio, and video) together with interactive functionalities to share information related to the study. If a digital consent solution does not have these capabilities, we suggest that it should be referred to as a digital consent form rather than a true eConsent solution.

### Limitations

Limitations of our systematic literature search include the fact that the search strategy that we used would have missed some potentially relevant studies while trying to keep the number of publications for screening manageable. Among eConsent studies, it is conceivable that there may be a reporting bias in favor of those finding comparative differences. Differences in the outcome measures used, including differences in their validity, made comparisons between studies challenging and pooling across studies unfeasible. Most studies included in this review did not provide a detailed description of the eConsent format, and such information should be included in future studies to allow researchers to assess and compare the results across studies. These observations are a call to action to harmonize the analysis, documentation, and reporting of eConsent findings, as well as the parameters defining best practices for eConsent (including whether these are met by current eConsent vendors). The same applies for the used terminologies and processes around eConsent. A current ongoing initiative of the European Forum of Good Clinical Practice aims to achieve the standardization within the clinical trial [69].

### Conclusions

In conclusion, this systematic review showed overall patient benefits with eConsent versus paper-based consenting in terms of understanding, acceptability, and usability. No study reported significantly better overall patient benefits with paper-based ICFs than with eConsent. eConsenting can increase enrollment into clinical studies by improving access to research. Comparative data from site staff and other study researchers indicate the potential for reduced workload and lower administrative burden with eConsent. In addition to these benefits, there are various other advantages associated with the use of digital solutions, including preventing flawed consenting processes, ensuring data quality, and supporting study integrity. Importantly, there are several avenues for future research that we believe are necessary. These include, but are not limited to, research that explores the best methodologies to target specific measures of eConsent efficacy (eg, recruitment, retention, and site experience); research that explores cross-cultural and globalization elements of eConsent; and research that makes better use of the theoretical underpinnings for why eConsent methods are more efficacious.

## Appendix

Multimedia Appendix 1 PRISMA (Preferred Reporting Items for Systematic Reviews and Meta-Analyses) checklist.

Multimedia Appendix 2 Overview of the studies identified for inclusion.

## Abbreviations

eConsent: electronic consent
FDA: Food and Drug Administration
ICF: informed consent form
PRISMA: Preferred Reporting Items for Systematic Reviews and Meta-Analyses
