# Supplementary material for: Comparative Effectiveness of eConsent: Systematic Review
Source: J Med Internet Res. 2023 Sep 1;25:e43883. doi: 10.2196/43883 (PMC10504628; doi:10.2196/43883)
Supplement: Multimedia Appendix 1 [file jmir_v25i1e43883_app1.docx]

| **Section and Topic** | **Item #** | **Checklist item** | **Location where item is reported** |
| --- | --- | --- | --- |
| **TITLE** | | |  |
| Title | 1 | Identify the report as a systematic review. | Title page |
| **ABSTRACT** | | |  |
| Abstract | 2 | See the PRISMA 2020 for Abstracts checklist. | Abstract Background, Objective, Methods, Results, Conclusions (see separate checklist below) |
| **INTRODUCTION** | | |  |
| Rationale | 3 | Describe the rationale for the review in the context of existing knowledge. | Introduction, paragraphs 1-3 |
| Objectives | 4 | Provide an explicit statement of the objective(s) or question(s) the review addresses. | Introduction, last paragraph |
| **METHODS** | | |  |
| Eligibility criteria | 5 | Specify the inclusion and exclusion criteria for the review and how studies were grouped for the syntheses. | Methods, “Inclusion and Exclusion Criteria” section |
| Information sources | 6 | Specify all databases, registers, websites, organisations, reference lists and other sources searched or consulted to identify studies. Specify the date when each source was last searched or consulted. | Methods, “Literature Searches” section |
| Search strategy | 7 | Present the full search strategies for all databases, registers and websites, including any filters and limits used. | Methods, “Literature Searches” section |
| Selection process | 8 | Specify the methods used to decide whether a study met the inclusion criteria of the review, including how many reviewers screened each record and each report retrieved, whether they worked independently, and if applicable, details of automation tools used in the process. | Methods, “Study Selection” section |
| Data collection process | 9 | Specify the methods used to collect data from reports, including how many reviewers collected data from each report, whether they worked independently, any processes for obtaining or confirming data from study investigators, and if applicable, details of automation tools used in the process. | Methods, “Data Collection and Summary” section |
| Data items | 10a | List and define all outcomes for which data were sought. Specify whether all results that were compatible with each outcome domain in each study were sought (e.g. for all measures, time points, analyses), and if not, the methods used to decide which results to collect. | Methods, “Data Collection and Summary” section |
|  | 10b | List and define all other variables for which data were sought (e.g. participant and intervention characteristics, funding sources). Describe any assumptions made about any missing or unclear information. | Methods, “Data Collection and Summary” and “Study Categorization” sections |
| Study risk of bias assessment | 11 | Specify the methods used to assess risk of bias in the included studies, including details of the tool(s) used, how many reviewers assessed each study and whether they worked independently, and if applicable, details of automation tools used in the process. | Data were analyzed descriptively (see Methods, “Data Collection and Summary” section). Studies comparing patient comprehension, acceptability, and usability were categorized by methodological validity (Methods, “Study Categorization” section). Risk of bias assessment was not conducted. |
| Effect measures | 12 | Specify for each outcome the effect measure(s) (e.g. risk ratio, mean difference) used in the synthesis or presentation of results. | Data were analyzed descriptively (see Methods, “Data Collection and Summary” section) |
| Synthesis methods | 13a | Describe the processes used to decide which studies were eligible for each synthesis (e.g. tabulating the study intervention characteristics and comparing against the planned groups for each synthesis (item #5)). | Data were analyzed descriptively (see Methods, “Data Collection and Summary” section) |
|  | 13b | Describe any methods required to prepare the data for presentation or synthesis, such as handling of missing summary statistics, or data conversions. | Data were analyzed descriptively (see Methods, “Data Collection and Summary” section) |
|  | 13c | Describe any methods used to tabulate or visually display results of individual studies and syntheses. | Data were analyzed descriptively (see Methods, “Data Collection and Summary” section) |
|  | 13d | Describe any methods used to synthesize results and provide a rationale for the choice(s). If meta-analysis was performed, describe the model(s), method(s) to identify the presence and extent of statistical heterogeneity, and software package(s) used. | Data were analyzed descriptively (see Methods, “Data Collection and Summary” section). No meta-analysis was performed. |
|  | 13e | Describe any methods used to explore possible causes of heterogeneity among study results (e.g. subgroup analysis, meta-regression). | Data were analyzed descriptively (see Methods, “Data Collection and Summary” section). Subgroup analyses or meta-regressions were not performed. |
|  | 13f | Describe any sensitivity analyses conducted to assess robustness of the synthesized results. | Data were analyzed descriptively (see Methods, “Data Collection and Summary” section). Sensitivity analyses were not conducted. |
| Reporting bias assessment | 14 | Describe any methods used to assess risk of bias due to missing results in a synthesis (arising from reporting biases). | Data were analyzed descriptively (see Methods, “Data Collection and Summary” section). Risk of bias die to missing results was not assessed. |
| Certainty assessment | 15 | Describe any methods used to assess certainty (or confidence) in the body of evidence for an outcome. | Methods, “Study Categorization” section |
| **RESULTS** | | |  |
| Study selection | 16a | Describe the results of the search and selection process, from the number of records identified in the search to the number of studies included in the review, ideally using a flow diagram. | Results, “Overview” section, first paragraph and Figure 1 |
|  | 16b | Cite studies that might appear to meet the inclusion criteria, but which were excluded, and explain why they were excluded. | Not applicable |
| Study characteristics | 17 | Cite each included study and present its characteristics. | Results, “Overview” section; References, Multimedia Appendix 2 |
| Risk of bias in studies | 18 | Present assessments of risk of bias for each included study. | Studies comparing patient comprehension, acceptability, and usability were categorized by methodological validity (Results, Tables 1-3). Risk of bias assessment was not conducted. |
| Results of individual studies | 19 | For all outcomes, present, for each study: (a) summary statistics for each group (where appropriate) and (b) an effect estimate and its precision (e.g. confidence/credible interval), ideally using structured tables or plots. | Findings were tabulated for studies comparing patient comprehension, acceptability, and usability (Tables 1-3). Data were analyzed descriptively. |
| Results of syntheses | 20a | For each synthesis, briefly summarise the characteristics and risk of bias among contributing studies. | Studies comparing patient comprehension, acceptability, and usability were categorized by methodological validity (Results, Tables 1-3). Risk of bias assessment was not conducted. |
|  | 20b | Present results of all statistical syntheses conducted. If meta-analysis was done, present for each the summary estimate and its precision (e.g. confidence/credible interval) and measures of statistical heterogeneity. If comparing groups, describe the direction of the effect. | Data were analyzed descriptively. Statistical syntheses or meta-analyses were not conducted. |
|  | 20c | Present results of all investigations of possible causes of heterogeneity among study results. | Data were analyzed descriptively. Results were overall consistent. Information on the different formats of eConsent used in the included studies is captured in the Results and discussed in a section on “Variability” in the Discussion. |
|  | 20d | Present results of all sensitivity analyses conducted to assess the robustness of the synthesized results. | Data were analyzed descriptively. Sensitivity analyses were not conducted. |
| Reporting biases | 21 | Present assessments of risk of bias due to missing results (arising from reporting biases) for each synthesis assessed. | Data were analyzed descriptively. Risk of bias due to missing results was not assessed. |
| Certainty of evidence | 22 | Present assessments of certainty (or confidence) in the body of evidence for each outcome assessed. | Studies comparing patient comprehension, acceptability, and usability were categorized by methodological validity (Results, Tables 1-3). Certainty of evidence assessment was not conducted. |
| **DISCUSSION** | | |  |
| Discussion | 23a | Provide a general interpretation of the results in the context of other evidence. | Discussion, “Effectiveness”, “Administration”, and “Variability” sections |
|  | 23b | Discuss any limitations of the evidence included in the review. | Discussion, “Limitations” section |
|  | 23c | Discuss any limitations of the review processes used. | Discussion, “Limitations” section |
|  | 23d | Discuss implications of the results for practice, policy, and future research. | Discussion, “Effectiveness”, “Administration”, “Variability”, “Limitations”, and “Conclusions” sections |
| **OTHER INFORMATION** | | |  |
| Registration and protocol | 24a | Provide registration information for the review, including register name and registration number, or state that the review was not registered. | Methods, “Literature Searches” section |
|  | 24b | Indicate where the review protocol can be accessed, or state that a protocol was not prepared. | Methods, “Literature Searches” section |
|  | 24c | Describe and explain any amendments to information provided at registration or in the protocol. | The review was not registered, and a protocol was not prepared (Methods, “Literature Searches” section) |
| Support | 25 | Describe sources of financial or non-financial support for the review, and the role of the funders or sponsors in the review. | “Acknowledgements”, “Authors’ Contributions”, and “Conflicts of Interest” sections |
| Competing interests | 26 | Declare any competing interests of review authors. | “Conflicts of Interest” section |
| Availability of data, code and other materials | 27 | Report which of the following are publicly available and where they can be found: template data collection forms; data extracted from included studies; data used for all analyses; analytic code; any other materials used in the review. | Not applicable. |

PRISMA for Abstracts

| **Section and Topic** | **Item #** | **Checklist item** | **Reported (Yes/No)** |
| --- | --- | --- | --- |
| **TITLE** | | |  |
| Title | 1 | Identify the report as a systematic review. | Y (Title page) |
| **BACKGROUND** | | |  |
| Objectives | 2 | Provide an explicit statement of the main objective(s) or question(s) the review addresses. | Y |
| **METHODS** | | |  |
| Eligibility criteria | 3 | Specify the inclusion and exclusion criteria for the review. | Y |
| Information sources | 4 | Specify the information sources (e.g. databases, registers) used to identify studies and the date when each was last searched. | Y |
| Risk of bias | 5 | Specify the methods used to assess risk of bias in the included studies. | Not applicable |
| Synthesis of results | 6 | Specify the methods used to present and synthesise results. | Y |
| **RESULTS** | | |  |
| Included studies | 7 | Give the total number of included studies and participants and summarise relevant characteristics of studies. | Y |
| Synthesis of results | 8 | Present results for main outcomes, preferably indicating the number of included studies and participants for each. If meta-analysis was done, report the summary estimate and confidence/credible interval. If comparing groups, indicate the direction of the effect (i.e. which group is favoured). | Y (Meta-analysis was not performed) |
| **DISCUSSION** | | |  |
| Limitations of evidence | 9 | Provide a brief summary of the limitations of the evidence included in the review (e.g. study risk of bias, inconsistency and imprecision). | Y (Some limited data noted; more details in main text) |
| Interpretation | 10 | Provide a general interpretation of the results and important implications. | Y |
| **OTHER** | | |  |
| Funding | 11 | Specify the primary source of funding for the review. | Y (Acknowledgements section) |
| Registration | 12 | Provide the register name and registration number. | Not applicable |

*From:*  Page MJ, McKenzie JE, Bossuyt PM, Boutron I, Hoffmann TC, Mulrow CD, et al. The PRISMA 2020 statement: an updated guideline for reporting systematic reviews. BMJ 2021;372:n71. doi: 10.1136/bmj.n71
