# Supplementary material for: Comparative Effectiveness of eConsent: Systematic Review
Source: J Med Internet Res. 2023 Sep 1;25:e43883. doi: 10.2196/43883 (PMC10504628; doi:10.2196/43883)
Supplement: Multimedia Appendix 2 [file jmir_v25i1e43883_app2.docx]

# Multimedia Appendix

## Multimedia Appendix 2

Overview of studies identified for inclusion.

| **Study (country)** | **Participants** | | | **Study design** | **Comparator method** | **Consent mode** | **Outcomes assessed** |
| --- | --- | --- | --- | --- | --- | --- | --- |
|  | **Population** | **N** | **Age, years** |  |  |  |  |
| Abujarad et al, 2021 [13] (USA) | Individuals eligible for an ongoing biorepository study, recruited at Chest Clinic and in the community | 50 | eConsent: mean 47 (SD^a^ 15) [range NR^b^]. Comparator: mean 38 (SD 15) [range NR] | Biorepository research study, subset of eligible participants randomized to multimedia vs paper-based consenting | Paper or interactive via tablet | In person, with remote preview | Comprehension, acceptability, usability, enrollment, cycle time |
| Afolabi et al, 2015 [14] (Gambia) | Adults eligible for inclusion in a malaria treatment trial | 311 | Mean, SD, range NR; >90% aged 18-49 | Randomized, controlled informed consent comprehension study with focus group discussions, nested within an RCT^c^ | Multimedia (DVD^d^) vs local standard (paper or verbal) | In person | Comprehension, acceptability, retention, cycle time |
| Bickmore et al, 2009 [15] (USA) | Participants recruited via fliers posted around university neighborhood and nearby apartment complex | 29 | Mean 60 (SD NR) [range 28-91] | Simulated consent study | Computer agent explanation vs research assistant explanation vs self-reading | In person | Comprehension, acceptability, usability, enrollment |
| Buckley et al, 2020 [16] (USA) | Patients who consented to biospecimen or genomic profiling-related protocols | 97 | NR | Follow-up of consented patients; survey of eConsenting platform users | Paper vs eConsent (details NR) | NA | Comprehension, acceptability, usability |
|  | Survey of patients using MSK^e^ eConsenting platform | 940 | NR |  |  |  |  |
| Cagnazzo et al, 2021 [17] (Italy) | Clinical research stakeholders (physicians, study coordinators, research nurses) | 99 | NR | Survey | Survey participants responded about paper vs electronic | NA^f^ | Acceptability, usability, enrollment, stakeholder view |
| Chalil Madathil et al, 2013 [18] (USA) | Patients were recruited for the study (details NR) | 40 | Mean NR (SD NR) [range 18-77] | Simulated consent study | Paper (existing consent form) vs electronic signature capture vs touchscreen vs iPad | In person | Comprehension, acceptability, usability, enrollment, cycle time, site workload, stakeholder view |
|  | Staff were recruited for the study (details NR) | 10 | Mean NR (SD NR) [range 23-74] |  |  |  |  |
| Chapman et al, 2021, 2020 [19, 20] (Australia) | Patients referred by a general practitioner to pathology services asked to participate in cardiovascular risk assessment study | 298 | Mean 63 (SD 8) [range 45-74] | Cardiovascular study, participants randomized to multimedia vs paper-based consenting | Paper vs multimedia | In person | Comprehension, acceptability, usability, cycle time |
| Fanaroff et al, 2018 [21] (USA) | Patients enrolling in a lipid-management study | 3485 | Median 68 [IQR^g^ 60-75] | Cholesterol management study conducted at 153 sites, of which 44% had approval to use video-based consenting | Sites using text-based consent vs sites approved to use video-based consent (both on tablet) | In person | Enrollment, retention, cycle time |
| Geier et al, 2021 [22] (USA) | Volunteer college students | 367 | Mean, SD, range NR; >98% aged 18-24 | Simulated consent study: online survey randomized participants to 1 of 6 variations of the consent form | Control (based on IRB^h^ template) vs interactive signature (“low interactive”) vs low reading level (4th grade) vs brevity (minimum information with option to view longer document) vs formatted (spaces with bold) vs page by page with questions at end of each page (“high interactive”) | NR | Comprehension |
| Golembiewski et al 2021 [23], Harle et al 2019 [24] (USA) | Patients making scheduled visits to their family medicine clinic were approached in clinic waiting rooms | 734 | Mean 46 (SD 16) [range NR] | Randomized, controlled informed consent study with immediate follow-up survey and additional follow-up phone calls at 1 week and 6 months | Standard (minimum information required by law) vs interactive (like standard, but with interactive hyperlinks on key terms) vs trust-enhanced (like interactive, but with information on regulations, researcher training and data protection) (all tablet-based) | In person | Comprehension, acceptability, enrollment, cycle time |
| Harmell et al, 2012 [25] (USA) | Outpatients with schizophrenia and healthy individuals, recruited through a registry | 35 | *Outpatients,* eConsent: mean 58 (SD 9). Comparator: mean 57 (SD 10). *Healthy individuals* eConsent: mean 49 (SD 16). Comparator: mean 53 (SD 12) [ranges NR] | Simulated consent study | Paper vs multimedia | In person | Comprehension, acceptability |
| Haussen et al, 2020 [26] (USA) | Legal authorized representatives of patients with large vessel occlusion stroke included in RCT | 53 | Median 63 [IQR 53-70] | eConsent for RCT in large vessel occlusion stroke; consenting via legal authorized representatives | Legal authorized representatives were asked if they would have preferred paper consent | Remote | Acceptability, usability |
| Jayasinghe et al, 2019 [27] (USA) | Community-dwelling individuals aged ≥65 years | 35 | Focus group: 77 (8) [range NR]. Pilot: 75 (7) [range NR] | Simulated consent study, consisting of focus group and randomized pilot study | Tablet vs paper | In person | Comprehension, acceptability, usability, cycle time |
| Jeste et al, 2009 [28] (USA) | Outpatients with schizophrenia and healthy individuals | 60 | Paper: mean 54 (SD 9). Multimedia: mean 55 (SD 7) | Simulated consent study | Paper (including a 10-min control video) vs multimedia (DVD) | In person | Comprehension, enrollment, cycle time |
| Jimison et al, 1998 [29] (USA) | Patients (recruited by clinical researchers); IRB members; researchers/experts in informed consent | 52 | NR | Focus groups, interviews | Follow-up focus groups and interviews after using a prototype multimedia tool included comments about how it compares with paper consenting | NA | Comprehension, acceptability, usability |
| Knapp et al, 2021 [49] (UK) | Adolescents attending orthodontic treatment | 109 | Median 13 [range 11-14] | Simulated consent for mock clinical trial, randomized | Paper vs multimedia | In person | Comprehension, usability, enrollment |
| McCarty et al, 2015 [30] (USA) | Individuals with prostate cancer enrolled in a population-based biobank | 56 | Mean 73 (SD NR) [range 55-86] | Randomized informed consent study with follow-up questionnaire at 6 months | Computer vs paper (for paper, researchers highlighted key elements and checked comprehension) | In person, with remote preview | Comprehension, usability, cycle time, site workload |
| McGowan et al, 2018 [31] (UK, Ireland) | Personnel returning from West African deployment | 111 | NR | Online consent for Ebola testing (via self-test kit); email with negative results included survey asking about online consent experience | Participants were asked if they would have preferred a face-to-face consent procedure | Remote | Comprehension, acceptability |
| McGraw et al, 2012 [32] (USA) | Patients visiting cancer clinics, general university community | 43 | Mean 38 (SD NR) [range 18-68] | Cognitive interviews with open-ended and debriefing questions | Paper vs multimedia | NA | Comprehension |
| Naeim et al, 2021 [33] (USA) | Hospital patients | 173 | Mean, SD, range NR; 21% aged <30, 78% aged 30-79 | Simulated consent (pilot testing) | Text-based video (phase 1) vs animated video (phase 2) | In person | Comprehension, usability, enrollment |
| Perrault et al, 2018 [34] (USA) | Undergraduate students | 547 | Mean 20 (SD 2) [range: 18-32] | Online simulated consent study: participants randomized to 1 of 7 different consent forms | Control (common consent form) vs spaces between lines vs spaces and key information bolded vs section headings/bullet points vs bullet points and bolded vs information in flowchart vs flowchart and bullet points | In person | Acceptability |
| Rothwell et al 2020 [35] (USA) | Parents deciding about consenting to storage and use of residual bloodspots from newborn screening | 669 | Mean 30 (SD 5) | Randomized education to aid informed consent to storage/use of screening samples, with follow-up surveys | Educational materials to aid in the consent process, delivered by brochure vs video vs interactive app | In person (but follow-up survey was remote) | Comprehension, enrollment |
| Rothwell et al, 2014 [36] (USA) | Pregnant women enrolling in a clinical trial | 62 | NR | Prenatal RCT of different educational materials, participants randomized to multimedia vs paper-based consenting | Standard (administered by nurse researcher, encouraged to read paper consent) vs eConsent (5-min video on iPad and paper consent) | In person | Comprehension, usability |
| Rowbotham et al, 2013 [37] (USA) | Clinical researchers, patients from a variety of clinical practices | 75 patients | Mean 50 (SD NR) [range 18-80] | Simulated consent study and follow-up survey | iPad vs paper | In person | Comprehension, acceptability, cycle time |
| Siegel et al, 2020 [38] (USA) | Patients with cancer | Phase 1: 342 (5 per month). Phase 2: 98 (65 per month) | NR | Consent for biobanking before and after redesign of consent process | Online consent before redesign (Phase 1; patients directed to consenting by email) and after redesign (Phase 2; interactive material added, video shortened; consenting integrated in new patient on-boarding) | Remote | Enrollment |
| Simon et al, 2016 [39] (USA) | Clinic patients and university community | 200 | Mean 47 (SD NR) [range 18-86] | Biobank recruitment process, patients randomized to different consenting formats | Face-to-face standard (paper consent with researcher); face-to-face enhanced (paper consent with researcher and targeted questions); multimedia standard (electronic slideshow with narration); enhanced interactive (electronic slideshow with narration, targeted questions) | In person | Comprehension, cycle time |
| Simon et al, 2018 [40] (USA) | Patients with hypertension; rural patients with and without hypertension | 50 | Mean 65 (SD NR) [range 43-82] | Focus group study | Paper vs electronic versions of IRB-approved informed consent for antihypertension trial | NA | Usability |
| Simon et al, 2021 [41] (USA) | Clinic patients | 501 | Mean 47 (SD NR) [range 18-84] | Biobank recruitment process, patients randomized to different consenting formats | Standard face-to-face vs eConsent (slides on tablet computer) with interactive questions (length, readability, and content of consenting materials differed by site [3 biobanks]). Researchers present for both conditions | In person | Comprehension, enrollment |
| Sonne et al, 2013 [42] (USA) | Individuals recruited on university campus | 61 | Mean 43 (SD 14) [range NR] | Simulated consent study | Paper vs video (all participants reviewed both formats and were randomized to which format they reviewed first) | In person | Comprehension, acceptability, cycle time |
| Tait et al, 2012 [43] (USA) | Individuals recruited from hospital waiting rooms | 9 | Adults: mean 44 (SD NR) [range 38-50]. Children: mean 11 (SD NR) [range 8-14] | Simulated consent study | Information on scores at baseline vs after interactive consent program | In person | Comprehension, acceptability |
| Vanaken et al, 2019 [44] (Multinational) | Center for Information and Study on Clinical Research Participation, Clariness, CenterWatch and TransCelerate participants; Health Authorities | Survey: 3045. Board: 10 | Survey: mean, SD, range NR; 84% aged ≥45. Board: mean NR (SD NR) [range 25-87] | Online survey, advisory board, health authority meetings | Survey question on preference for paper vs electronic | NA | Comprehension, acceptability, site workload, stakeholder view |
| Varnhagen et al, 2005 [45] (Canada) | University students | 100 | NR | Simulated consent study, randomized | Online vs paper (both either continuous [long] or paginated) | In person | Comprehension, cycle time |
| Vercauteren et al, 2020 [46] (Canada) | Biobank participants and patient network | 113 | Mean, SD, range NR; 27% aged ≤18 years | Survey | Survey question on preference for paper vs electronic | NA | Acceptability |
| Warriner et al, 2016 [47] (USA) | Women taking alendronate (also included a satisfaction survey of practice site physicians/staff) | 33 | Tablet: mean 69 (SD 7). Paper: mean 71 (SD 9) [ranges NR] | Simulated consent for mock clinical trial, randomized | Tablet (iPad) vs paper | In person | Comprehension, acceptability, cycle time, stakeholder view |
| Zeps et al, 2020 [48] (Australia) | Clinical trial researchers | 179 | Mean, SD, range NR; 75% aged ≥40 years | Survey, interviews | Participants were asked about their perceptions on the use of eConsent vs paper consent | NA | Comprehension, acceptability, site workload, stakeholder view |

^a^SD: standard deviation.

^b^NR: not reported.

^c^RCT: randomized controlled trial.

^d^DVD: digital versatile/video disk.

^e^MSK: Memorial Sloan Kettering.

^f^NA: not applicable.

^g^IQR: interquartile range.

^h^IRB: institutional review board.
